# Computational Simulations of Fabrication of Aluminum-Based Josephson Junctions: Topological Aspects of the Barrier Structure

**DOI:** 10.3390/e25020182

**Published:** 2023-01-17

**Authors:** Chuanbing Han, Huihui Sun, Fudong Liu, Xiangju Zhao, Zheng Shan

**Affiliations:** 1 State Key Laboratory of Mathematical Engineering and Advanced Computing, Zhengzhou 450001, China; 2 National Supercomputing Center in Zhengzhou, Zhengzhou University, Zhengzhou 450001, China

**Keywords:** Josephson junction, molecular dynamics, film growth, topological analysis

## Abstract

Although the performance of qubits has been improved in recent years, the differences in the microscopic atomic structure of the Josephson junctions, the core devices prepared under different preparation conditions, are still underexplored. In this paper, the effects of the oxygen temperature and upper aluminum deposition rate on the topology of the barrier layer in the aluminum-based Josephson junctions have been presented by classical molecular dynamics simulations. We apply a Voronoi tessellation method to characterize the topology of the interface and central regions of the barrier layers. We find that when the oxygen temperature is 573 K and the upper aluminum deposition rate is 4 Å/ps, the barrier has the fewest atomic voids and the most closely arranged atoms. However, if only the atomic arrangement of the central region is considered, the optimal rate of the aluminum deposition is 8 Å/ps. This work provides microscopic guidance for the experimental preparation of Josephson junctions, which helps to improve the performance of qubits and accelerate the practical application of quantum computers.

## 1. Introduction

Quantum computing [1] is a new computing solution that follows the laws of quantum mechanics and can perform high-speed calculation, storage, and processing of information. Compared to traditional classical computers, quantum computers have natural quantum parallel computing capabilities, which will bring a qualitative leap to existing computing capabilities. The research on quantum computing is a scientific attempt to break through the limits of classical computing power, and it has now become a scientific and technological means with which countries can seize the future [2]. As one of the most promising candidate technology routes for building a universal quantum computer, the performance of the core device of superconducting qubits [3,4]—Josephson junctions [5,6]—is crucial.

The experimental Josephson junction is usually made of an Al/AlO*_x_*/Al trilayer structure, and the aluminum oxide Josephson junction is currently the best-developed junction for quantum computers [7,8]. The barrier in the junction is usually only 2 nm thick, and its morphology and interface with the superconducting layer have a significant impact on the coherence time and performance of qubits. The importance of the Josephson junction simulations has been demonstrated using a variety of theories [9,10], in particular the atomic scale simulations that show that the structure and nanochemical characteristics of the AlO*_x_* barrier are closely related to manufacturing conditions.

The simulations also confirm that the morphological evolution and roughness of oxides formed by different crystal orientations are very different and show the obvious differences in the local atomic environment related to the short and medium ranges [11,12,13,14]. The self-limiting reaction found in experiments [15,16] (when the oxidation temperature is greater than 573 K, the thickness of the oxide is no longer restricted) can be explained by simulations [11,12,13], i.e., as the oxidation temperature increases, the number of O-O bonds on the outermost Al surface decreases, resulting in the formation of a thicker and more disordered oxide film. Similarly, oxidation time has been studied in experiments [16,17,18] and simulations [13,14]. Furthermore, oxidation pressure is experimentally studied [19,20], and oxygen density in the simulations [11] is studied. It has been experimentally found that the oxidation mechanisms of different Al substrate crystal orientations are different [21,22]. The film undergoes a distinctly different structural evolution during growth. It is worth noting that the epitaxial Al layer can be experimentally found to be realized on Si(111) at a low substrate temperature and a high deposition rate [23]. However, no systematic studies of deposition rates have been carried out, either in experiments or simulations during the preparation of Josephson junctions. Moreover, the composite effect of multiple parameters on the structures of Josephson junctions has been poorly studied.

In this paper, we construct an aluminum oxide Josephson junction preparation model using classical molecular dynamics simulations to investigate the effects of the oxygen temperature and the upper aluminum deposition rate on the topology of the barrier layer. We divide the central region of the barrier layer and the interface region between the barrier and the superconducting layer by the positions of oxygen atoms. Using two structural descriptors, atomic volume and configuration entropy, we conclude that when the deposition rate is 8 Å/ps, we can more tightly pack the atoms of the central region. When the oxygen temperature is 573 K and the upper aluminum deposition rate is 4 Å/ps, the barrier layer with tightly arranged particles in the interface and central regions can be prepared by simulations.

## 2. Methods

### 2.1. Molecular Dynamics

The unit cell parameter of the lower crystalline aluminum is 4.041386 Å, which is rendered from experiments [24]. It consists of 4×4×10 crystal cells, containing 672 aluminum atoms in the aluminum substrate. The periodic boundary conditions are introduced in the x- and y-directions. The velocity-Verlet algorithm is applied to integrate the equation of motion, with the timestep of 1 fs. The aluminum substrate is divided into three regions along the *z*-axis, including the fixed-region, thermostatic-region, and free-region. To avoid system drift, the fixed-region is composed of three layers of atoms at the bottom of the aluminum substrate. The Nosé–Hoover thermostat [25,26] is used in the thermostatic-region to provide approximate isothermal conditions for the system and absorb the additional energy generated by the reaction in the free-region. The temperature of the thermostatic-region corresponds to the variable $T_{substrate}$. At the top of the substrate, in the free-region, four layers of atoms allow for unfettered particle-to-particle bonding.

**Figure 1:**
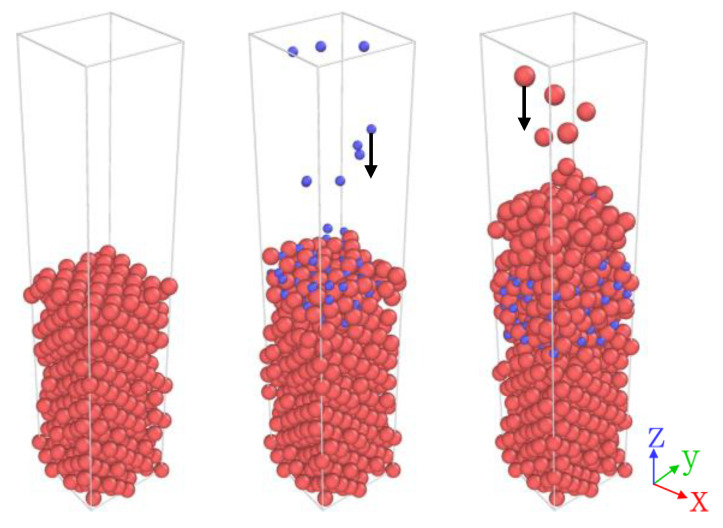
Simulation process diagrams of the fabrication of the aluminum oxide Josephson junction. The first subplot is the initial aluminum substrate model. The second and third subplots show the oxidation process and the upper aluminum deposition process, respectively. Oxygen and aluminum atoms are represented by blue and red spheres, respectively.

The double-angle evaporation process [27] is the most common method to produce high-quality trilayer Al-AlO*_x_*-Al Josephson junctions. Aluminum layers are deposited on the substrate at different angles through a photolithographic mask, and an oxide barrier layer is formed by intermediate low-pressure oxidation [28]. This process involves two critical parameters, the deposition rate of oxygen and aluminum atoms, as shown in Figure 1.

Since experimental junctions are usually made under high vacuum and the partial oxygen pressure is usually low pressure (10^−12^–10^−2^ atm) [20], we believe that oxygen is subject to the ideal gas law equation [13]. In this case, we define the distribution of oxygen velocity f(v) to satisfy the Maxwell–Boltzmann distribution, which is defined as

(1) $$f(v)={\left(\frac{m}{2\pi k_{B}T}\right)}^{3/2}4\pi v^{2}\exp(-\frac{mv^{2}}{2k_{B}T})$$

v is the velocity of oxygen; m is the mass of oxygen; $k_{B}$ is Boltzmann constant; and T is temperature.

In this paper, the Gaussian distribution is used to determine the oxygen atoms’ initial velocity, with mathematic expectation $u=\sqrt{\frac{8k_{B}T_{oxygen}}{\pi m}}$ and standard deviation $\sigma =\sqrt{\frac{k_{B}T_{oxygen}}{\pi m}}$, where $T_{oxygen}$ is set to the oxygen temperature and constrained to be directed towards the aluminum substrate surface. Then, the system evolves for 12 ps. The method of upper aluminum deposition is the same as that of oxidation, and the velocity is defined as $v_{deposit}$. All of the deposited atoms are placed at the top 0.5 Å of the simulated box with random positions of x and y. In our simulations, the number of oxygen atoms deposited at 77 K is 118, while around 160 oxygen atoms are required at other temperatures. The final stage requires the deposition of approximately 288 aluminum atoms.

DuBois et al. used the Streitz–Mintmire (S-M) potential [29] to capture the variable oxygen charge state and found that TLS defects exist at the two metal–oxide interfaces in the junction [30], which greatly affects the performance of JJ. Therefore, in this paper, the S-M potential is used to describe the interaction between aluminum and oxygen atoms. To further reveal the connection between the topology of the aluminum oxide barrier and the deposition rate, we designed several groups of simulation experiments, the key parameters of which are listed in Table 1. The temperature of liquid nitrogen cooling (77 K), the common oxidation temperature (298 K), and the critical temperature of aluminum oxidation about the self-limiting reaction (573 K) [15] are chosen for interest in the experiments. All simulations are carried out using LAMMPS [31,32]. The open-source tool OVITO [33] is used to visualize the simulation process.

### 2.2. Voronoi Tessellation and Coordination Number

Voronoi tessellation divides space into tightly packed polyhedrons through the local structure between atoms and their neighboring atoms. More information on Voronoi tessellation methods can be found in Refs. [34,35]. Taking into account the radii of particles, we apply poly-disperse Voronoi tessellation. The Voronoi index $<n_{3},n_{4},n_{5},n_{6}>$, with $n_{i}$ denoting the number of faces with *i*-edged, can be viewed as a characteristic signature of the coordination structure of a particle and the topology of the Voronoi polyhedron (VP). Additionally, $\sum_{i}n_{i}$ is usually called the coordination number.

## 3. Results and Discussion

### 3.1. Distribution of Voronoi Polyhedron Atomic Volume

**Figure 2:**
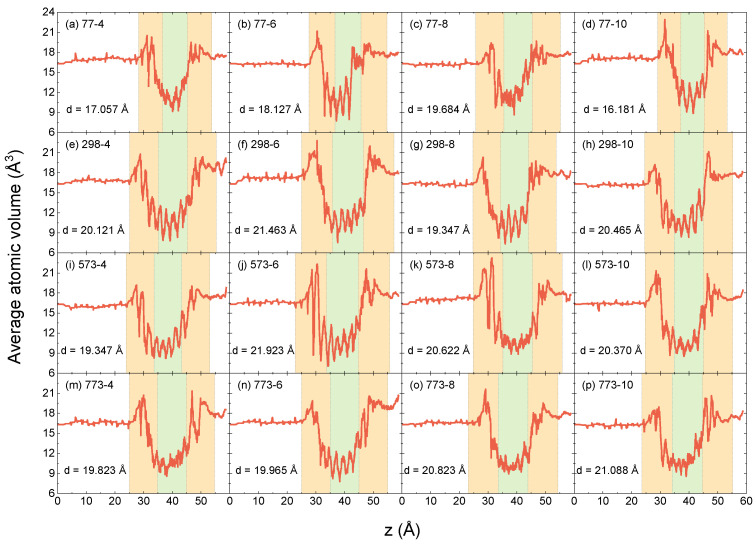
Average atomic volume profiles along the *z*-axis for various oxygen temperatures and aluminum deposition rates. The subheading *i*-*j* indicates that the oxygen temperature is *i* K and the aluminum deposition rate is *j* Å/ps. For example, 77-4 represents the oxygen temperature of 77 K and the deposition rate of 4 Å/ps.

The oxide thickness d in the aluminum-based tunnel junction in this paper is determined by the positions of the outermost oxygen atom $O_{L}$ and $O_{R}$ along the *z*-axis, i.e., $d=O_{R}-O_{L}$. The three regions divided by four values $\left(O_{L}-d/4,O_{L}+d/4,O_{R}-d/4,O_{R}+d/4\right)$ are defined as the lower-interface, central, and upper-interface regions, where the thickness of each region is d/2. We calculate the distribution of the average atomic volume along the *z*-axis of the AlO*_x_* Josephson junction models prepared at different parameters, as shown in Figure 2. The three regions are distinguished by orange and green colors. The oxide thickness for different parameters is listed in the lower-left corner of each subplot in Figure 2. It can be seen that when the oxygen temperature is 77 K, the thickness of the barrier layer is the smallest, mostly below 2 nm, which is consistent with our previous work [36]. Atoms in the aluminum superconducting layer are arranged in order, and the average atomic volume fluctuates around 16.5 Å^3^.

For the central region, the atomic volume fluctuates between 7.5 and 15 Å^3^, but the volume distribution is not very regular at different oxygen temperatures and deposition rates. We performed standard deviation statistics for the sampling points in each region and obtained the fluctuation of the atomic volume in each region. Compared to other rates, the fluctuation in atomic volume in the central region of the oxide obtained at 8 Å/ps was minimal (the standard deviation fluctuates from top to bottom in the third column of Figure 2 is 2.223, 1.579, 0.834, and 0.955), indicating that regardless of the oxygen temperature, while 8 Å/ps appears to be the optimal deposition rate of aluminum for the central region to obtain the closed-packing atoms. The oxygen temperature does not seem to have a significant effect on the distribution of atomic volume in the central region.

We argue that the deposition rate of the upper layer of aluminum has little effect on the atomic arrangement in the lower-interface region because if the barrier layer is thick, the aluminum electrode prepared above will not affect the lower-interface region. However, if the thickness of the barrier layer is less than 2 nm, it is very sensitive to external conditions, so the deposition rate of the upper aluminum will also affect the tightness of the lower-interface region to some extent. Additionally, by comparing the four subgraphs in each row in Figure 2, this can also be proved.

**Figure 3:**
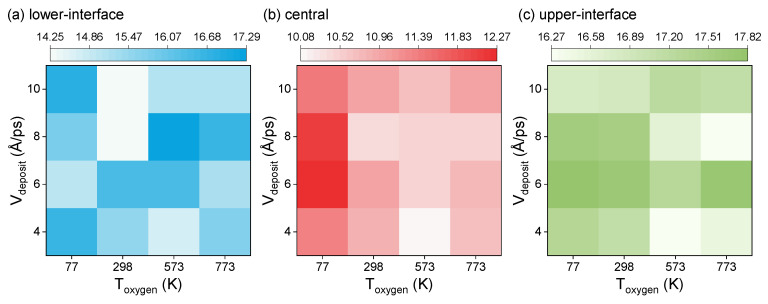
Average atomic volume of (**a**) lower-interface, (**b**) central, and (**c**) upper-interface regions of the barrier layer at various oxygen temperatures and aluminum deposition rates.

We calculate the average atomic volume of all atoms in the three regions, respectively, as shown in Figure 3. For an ideal Josephson junction, to improve the performance of qubits (a better T1 lifetime), its atoms need to be closely arranged, that is, the atomic volume is small. In Figure 3a, we can see that the particles are arranged more closely under the three groups of parameters (298-10, 298-8, and 573-4), with average atomic volumes of 14.274, 14.252, and 14.680 Å^3^, respectively. The average atomic volume under the three groups of parameters (298-8, 773-8, and 573-4) in Figure 3b is small, which is 10.383, 10.518, and 10.087 Å^3^, respectively, while the average atomic volume under the parameters of 773-8 and 573-4 in Figure 3c are 16.279 and 16.267 Å^3^, respectively. Therefore, when the oxygen temperature is 573 K and the aluminum deposition rate is 4 Å/ps, the average atomic volume of the barrier interface and central regions of the Josephson junction is smaller, so that the atoms are more compact. We also find that when the aluminum deposition rate is 6 Å/ps, the atomic volume fluctuates less at different oxygen temperatures, which seems to explain why the average aluminum deposition rate in the evaporation method of film deposition commonly used in experiments is 600 m/s [37].

### 3.2. Distribution of Configurational Entropy

We analyze the order of the materials using the configuration entropy based on the Voronoi index to characterize the structure order of the interface and central regions of the thin films formed under various parameters. The configuration entropy $S_{conf}$ [38] can be calculated by the following formula:

(2) $$S_{conf}=-R*\sum (V*\ln(V))$$

where V is the probability of a certain coordination number and R is the gas constant.

**Figure 4:**
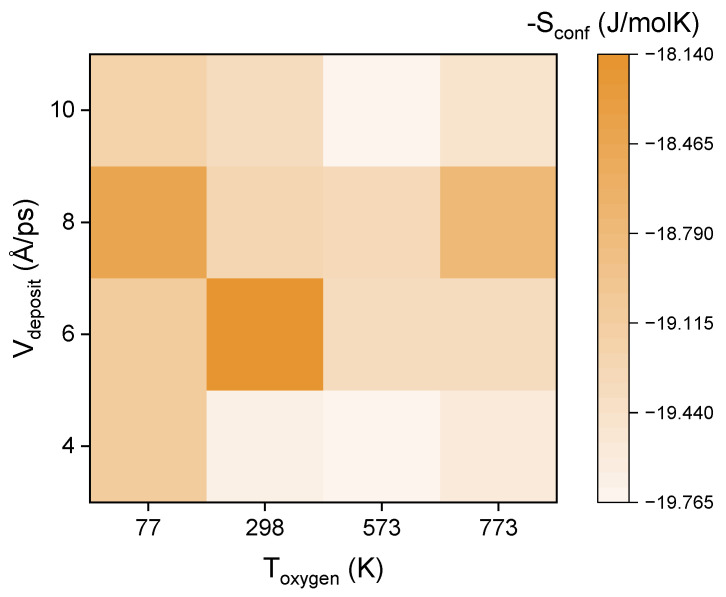
Configurational entropy at different oxygen temperatures and aluminum deposition rates.

Considering the coordination number mentioned in Equation (2), we calculate the Voronoi index of each atom under each group of parameters and obtain the coordination number and its proportion according to the method in Section 2.2. The overall configuration entropy of the interface and the central regions of the barrier layer under each group of parameters is shown in Figure 4. If there is a large void space in the barrier, the atoms may be delocalized, leading to B-type Two-Level System (TLS) defects [30]. TLS defects are one of the major challenges [8] that need to be overcome and avoided for the preparation of the oxide Josephson junction, and their appearance will reduce the performance of thin films and the coherence time of qubits. Therefore, according to the analysis of Figure 4, the barrier layer prepared at the oxygen temperature 573 K and the aluminum deposition rate 4 Å/ps has a higher performance.

In this paper, the velocity direction of all of the deposited atoms is limited to being directed towards the surface of the aluminum substrate, which is not in line with the actual meaning of the oxidation temperature in the experiments. Therefore, in our future work, we should try to make the velocity direction of the deposited atom random, pointing in any direction, so as to match the real experimental conditions as closely as possible.

## 4. Conclusions

The behavior of some macroscopic properties of qubits, such as the lifetime T1, engages with the atomic arrangement of the core device—Josephson junctions—especially with the interface and central regions of the barrier layer. In this paper, we use molecular dynamics simulations to simulate the complete preparation process of the aluminum oxide Josephson junctions. Moreover, we use the Voronoi tessellation method to study the effects of the oxygen temperature and the upper aluminum deposition rate on the topology of the interface and central regions of the barrier layer. The thicknesses of the barrier layer made under different parameters are all less than 2.2 nm, and the thickness of AlO*_x_* at the oxygen temperature 77 K is less than 2 nm. By comparing the atomic volumes, we find that the deposition rate of 8 Å/ps can obtain the central region with the tightly arranged atoms. However, considering the interface region, we compare the atomic spatial structure of each region from the perspective of atomic volume and configuration entropy, and we believe that when the oxygen temperature is 573 K and the upper aluminum deposition rate is 4 Å/ps in the experiment, it is possible to obtain a barrier layer with atoms closely arranged in the interface and central regions.

## Figures and Tables

**Table 1:** Simulation parameters. (4 * 4 = 16 groups in total).

$T_{substrate}\;(K)$	$T_{oxygen}\;(K)$	$v_{deposit}\;(\text{Å}/\mathrm{ps})$
298	77	4
	77	6
	77	8
	77	10
	298	4
	298	6
	298	8
	298	10
	573	4
	573	6
	573	8
	573	10
	773	4
	773	6
	773	8
	773	10

## Data Availability

The data that support the findings of this study are available from the corresponding author upon reasonable request.
